# Predictors of Retention in an Adult Text Messaging Smoking Cessation Intervention Program: Cohort Study

**DOI:** 10.2196/13712

**Published:** 2019-08-01

**Authors:** Kara P Wiseman, Kisha I Coa, Yvonne M Prutzman

**Affiliations:** 1 Tobacco Control Research Branch Behavioral Research Program National Cancer Institute Bethesda, MD United States; 2 ICF Fairfax, VA United States

**Keywords:** smoking cessation, text-messaging, mHealth, engagement

## Abstract

**Background:**

Mobile health tools such as text messaging programs can support smoking cessation. However, high rates of disengagement from these tools decrease their effectiveness.

**Objective:**

The purpose of this study was to identify user characteristics associated with retention in an adult text messaging smoking cessation intervention.

**Methods:**

Adults initiating a quit attempt using the publicly available program SmokefreeTXT between March 6 and June 21, 2016 (n=6215), were included. Data were collected to assess nicotine dependence, frequency of being around other smokers, time of the day for cigarette cravings, extrinsic and intrinsic motivation to quit smoking, confidence in quitting, and long-term intention to be smoke free. Multivariable survival analysis modeling for time to opt out was conducted to identify characteristics associated with opting out over the course of the intervention, adjusting for age, sex, and smoking frequency, reset of the quit date by the user, and the number of days enrolled before initiating the quit attempt. Among those who opted out, multivariable multinomial logistic regression analysis was used to identify predictors of opting out early (within 3 days and between 4 and 7 days into the quit attempt) compared to opting out late (more than 7 days into the quit attempt), adjusting for the same confounders.

**Results:**

Survival analyses indicated that younger age, female sex, higher levels of nicotine dependence, lower intention to be smoke free, and enrolling in SmokefreeTXT ≤1 week before initiating the quit attempt were associated with an increased risk of opting out. For example, users who smoked within 5 minutes of waking up were 1.17 times more likely to opt out than those who smoked more than 5 minutes after waking up (95% CI 1.01-1.35). Among users who opted out from SmokefreeTXT, logistic regression modeling indicated that compared to users who were never or rarely around other smokers, those who were sometimes around other smokers had 1.96 times more likely to opt out within the first 3 days of the quit attempt (95% CI 1.18-3.25). In addition, compared to users with high levels of long-term quit intention, users with lower levels of intention had 1.80 times the odds of opting out between 4 and 7 days into the quit attempt (95% CI 1.02-3.18). Users who reset their quit date after initiating a quit attempt were less likely to opt out at either time point compared with those who did not reset their quit date.

**Conclusions:**

Several user characteristics are associated with retention in an adult text messaging smoking cessation program. These results provide guidance on potential characteristics that should be addressed in future text messaging smoking cessation programs. Providing additional support to users with these characteristics may increase retention in text messaging programs and ultimately lead to smoking cessation.

## Introduction

Although smoking rates continue to decline, smoking is responsible for more than 480,000 deaths annually in the United States [1]. Mobile health (mHealth) smoking cessation programs are a cost-effective way to reach many smokers, particularly smokers who are underserved by traditional treatment modalities (eg, in-person or telephone counseling) [2,3]. Text messaging smoking cessation programs have been found to as much as double a smoker’s likelihood of quitting [4,5]; however, high user dropout rates reduce the potential benefit of these programs [6,7]. A recent study found that smokers who left a text-based cessation program were less likely to abstain from smoking at 6 months posttreatment compared with those who remained in the program for longer [8], suggesting that increasing retention in a cessation program may increase the likelihood of quitting.

There are many potential reasons for someone to leave a cessation program prior to program completion, including failure to initiate a quit attempt or relapse, low initial motivation to quit, a high level of nicotine dependence and withdrawal symptoms, inability to resist cravings, or lack of sufficient social support during a quit attempt [9,10]. Text-based cessation programs have the potential to address some of these issues, but it is still unclear which users are more likely to leave a cessation program, and to date, the most relevant characteristics related to opting out of text messaging cessation programs have not been identified [4]. A better understanding of the characteristics associated with opting out may illuminate potential avenues for program enhancement and help identify the types of program users who may benefit most from new or additional program content.

The purpose of this study was to determine whether potentially relevant and addressable user characteristics measured during program enrollment were associated with opting out from SmokefreeTXT, one of the most widely used text-based smoking cessation programs available in the United States.

## Methods

### SmokefreeTXT Cessation Program

SmokefreeTXT is a free, nationally available, fully automated text-messaging smoking cessation program for adults run by the National Cancer Institute [7]. Smokers interested in quitting smoking can sign up for the program using a Web enrollment form or short message service (text) opt in. The program includes up to 2 weeks of preparation messages and 6 weeks of postquit date messages, and messages vary in content and frequency relative to the quit date set by the user. Text messages provide general motivation support, tips on preparing to quit, advice on managing cravings, suggestions for smoke-free activities, relevant smoking facts, and recognition of cessation milestones [7]. The program is bidirectional; users can text keywords (ie, “crave,” “slip,” or “mood”) at any time to receive on-demand support. Self-reported smoking status, mood, and craving levels are measured using within-program assessment questions delivered during the program. Users can reset their quit date at any time (eg, after a slip) to restart the program. Users can opt out of the program at any time by texting “STOP.” Since the program began in 2011, over 150,000 people have enrolled in SmokefreeTXT [11].

### Data Collection and Study Population

#### Design and Measures

Data on age, sex, and smoking frequency are routinely collected from all SmokefreeTXT users at the time of program enrollment. For this study, eight additional baseline items, adapted from validated scales when available, were added to examine smoking context and motivational characteristics associated with opting out. The smoking context characteristics measured the level of nicotine dependence (measured as time to first cigarette: “How soon after you wake up do you smoke your first cigarette?” [12]), frequency of reminders to smoke (“My life is full of reminders to smoke” [13]), time of the day for cigarette cravings (“When do you crave cigarettes the most?” [13]), and frequency of being around other smokers (“How often are you around people who are smoking?”). The motivational characteristics measured extrinsic motivation for quitting smoking (“I would try to quit smoking because others want me to quit smoking” [14]), intrinsic motivation for quitting smoking (“I would try to quit smoking because quitting smoking is an important thing for me to do” [14]), confidence in quitting (“I feel able to meet the challenge of quitting smoking”), and long-term cessation intention (“I intend to be smokefree one year from now” [15]). To minimize respondent burden, only single-item measures were used, and each SmokefreeTXT user received only two of the eight additional questions at the time of enrollment. Specifically, with each page refresh, two items were randomly selected from the bank of eight items for inclusion on the Web enrollment form. Under the National Institutes of Health policy, assessment of quality improvement processes does not require institutional review board approval and is covered by the Terms of Agreements that users agree to when signing up for the SmokefreeTXT program [16]. All data were nonidentifiable, and unique user identifiers replaced user telephone numbers before data were received from the text-message program vendor.

#### Study Population

The study population consisted of the first quit attempt of all users who signed-up for SmokefreeTXT using the Web enrollment form between March 6, 2016, and June 21, 2016 (N=6831 unique users). Of these, 616 were excluded (571 opted out of SmokefreeTXT before reaching their initial quit date, 41 set quit dates outside of the 2-week window [range: 46 days until the initial quit date to 2 days after a quit date], and 4 had unreliable ages [eg, age >99 years]), leaving 6215 users in the analytic sample.

### Analysis

#### Coding

##### Opting Out of the Program

Two outcome variables were created to measure the timing for opting out of the program. The date and time any user texts “STOP” to opt out are saved, as are the date and time of enrollment. The number of days that a user was enrolled before opting out was determined by calculating the difference between a user’s initial quit date and the date of opting out. Users can reset their quit date by texting “NEW” to SmokefreeTXT. Receipt of this message is date and time stamped and when this occurs, a new row of data is created to capture program data relative to the new quit date. If users reset their quit date within the program, the program data capturing their most up-to-date quit date were used to determine timing of opting out. For users who did not opt out, time to opt out was censored at 42 days (length of the full 6-week intervention starting on the quit date). Previous findings show that the majority of users opting out of SmokefreeTXT do so in the first 3 days and up to the first week after initiating their quit attempt [7]; therefore, to understand the user characteristics associated with opting out early compared to opting out later in the program, a three-level variable was created among users who opted out of the program (n=3259) in order to investigate opting out within 3 days after the initial quit date and opting out between 4 and 7 days after the initial quit attempt compared with opting out more than 7 days after the initial quit date.

##### Characteristics of Interest

The eight baseline items added to the Web enrollment form were the primary characteristics of interest for this study and were coded using the distribution of responses in the study population. These characteristics were level of nicotine dependence (two levels: smokes within 5 minutes of waking up and smokes more than 5 minutes after waking), frequency of reminders to smoke (two levels: very true and a little true or a little/very untrue), time of the day for cigarette cravings (two levels: craves cigarettes in the morning, afternoon, or evening and craves cigarettes the same amount all times of day), frequency around other smokers (three levels: never or rarely around other smokers, sometimes around other smokers, and very often around other smokers), extrinsic motivation for quitting smoking (three levels: very true, a little true, and a little/very untrue), intrinsic motivation for quitting smoking (two levels: very true and a little true or a little/very untrue), confidence in quitting (three levels: very true, a little true, and a little/very untrue), and long-term cessation intention (two levels: strongly agree and agree or disagree/strongly disagree).

##### Potential Confounders

Information collected from users at the time of program enrollment were considered as potential confounders: age (18-29, 30-39, 40-49, and ≥50 years), sex (male or female), and smoking frequency (smokes every day or smokes less often than every day). Two additional characteristics were also included. First, a variable was created to indicate if a user had ever reset their quit date (ie, yes [used the keyword “NEW” coded as yes] or no). Lastly, the number of days that a user was enrolled before starting their cessation attempt was included to capture receipt of cessation preparation messages (0, 1-7, and 8-14 days).

#### Statistical Analyses

SAS 9.4 (SAS Institute, Cary NC) was used for all analyses. Descriptive analyses were performed using Chi-square tests. Multivariable survival analysis modeling for days to opt out was used to determine user characteristics associated with an increased risk of opting out. For all models, an initial confounder-only model (age, sex, smoking frequency, reset of the quit date by the user, and days enrolled before starting the quit attempt) was created. Thereafter, eight separate models were created with each independent variable of interest. Due to the data collection design, each user only answered two of the eight items of interest, with each item being randomly pulled from the bank of the eight items; thus, information on all eight items is not available for each user, and multiple independent variables of interest could not be included in the same model. Frequency around other smokers had a violation of the proportional hazards assumption; further investigation revealed that the assumption was violated at 14 days after the quit date; thus, two models were created for this user characteristic—one for opting out in the first 14 days and one for opting out after 14 days. Models provided adjusted hazard ratios and 95% CIs. Among users who opted out, multivariable multinomial logistic regression modeling was performed to examine the association between each user characteristic and opting out within 3 days (or between 4 and 7 days) compared to opting out after 7 days. Again, an initial confounder-only model was created and each user characteristic of interest was added to the confounder-only model. Logistic regression models provided odds ratios and 95% CIs.

## Results

Overall, the majority of SmokefreeTXT users were women (69.4%), smoked every day (92.4%), had frequent reminders to smoke (68.7%), and craved cigarettes at all times of the day (67.1%) (Table 1). Most users reported high extrinsic and intrinsic motivation for quitting smoking (43.1% and 91.2% reported very true, respectively). Just over one-third of the users signed up for the program on their quit date (37.5%). In addition, 81% of users did not reset their quit date during the program. Slightly more than half (52.4%) opted out during the 6-week course of the program. User characteristics by the timing of the opt-out variable can be found in Multimedia Appendix 1.

**Table 1:** Descriptive characteristics of SmokefreeTXT users from March 3, 2016, to June 21, 2016.

Variable		Total, n (%)	Opted out during the program		*P* value^a^
			Yes, n (%)	No, n (%)	
Number of users		6215 (100)	3259 (52.4)	2956 (47.6)	
**Age (years)**					<.001^b^
	18-29	1823 (29.3)	1052 (32.3)	771 (26.1)	
	30-39	1823 (29.3)	957 (29.4)	866 (29.3)	
	40-49	1288 (20.7)	659 (20.2)	629 (21.3)	
	≥50	1281 (20.6)	591 (18.1)	690 (23.3)	
**Sex**					.009^b^
	Male	1904 (30.6)	951 (29.2)	953 (32.2)	
	Female	4311 (69.4)	2308 (70.8)	2003 (67.8)	
**Smoking frequency^c^**					.16
	less often than every day	468 (7.6)	231 (7.2)	237 (8.1)	
	Every day	5672 (92.4)	2992 (92.8)	2680 (91.9)	
**Time to first cigarette (min)^d, e^**					.13
	>5	1025 (62.7)	511 (60.9)	514 (64.6)	
	≤5	610 (37.3)	328 (39.1)	282 (35.4)	
**Frequent reminders to smoke^d^**					.90
	Not true^f^	472 (31.3)	252 (31.5)	220 (31.2)	
	Very true	1035 (68.7)	549 (68.5)	486 (68.8)	
**Frequency around other smokers^d^**					.13
	Never or rarely	295 (18.8)	145 (18.1)	150 (19.5)	
	Sometimes	506 (32.2)	247 (30.8)	259 (33.7)	
	Very often	771 (49.1)	411 (51.2)	360 (46.8)	
**Craves cigarettes at a specific time of day^d^**					.46
	No	1023 (67.1)	536 (67.9)	487 (66.2)	
	Yes	502 (32.9)	253 (32.1)	249 (33.8)	
**Extrinsic motivation to quit^d^**					.56
	Very true	685 (43.1)	347 (42.5)	338 (43.8)	
	A little true	502 (31.6)	259 (31.7)	243 (31.5)	
	A little or very untrue	402 (25.3)	211 (25.8)	191 (24.7)	
**Intrinsic motivation to quit^d^**					.19
	Not true^f^	134 (8.8)	79 (9.7)	55 (7.8)	
	Very true	1392 (91.2)	739 (90.3)	653 (92.2)	
**Confidence in quitting smoking^d^**					.35
	A little or very untrue	295 (19.0)	163 (19.6)	132 (18.4)	
	A little true	652 (42.0)	354 (42.5)	298 (41.5)	
	Very true	605 (39.0)	316 (37.9)	289 (40.2)	
**Long-term quit intention^d, g^**					.21
	Other responses^h^	182 (12.6)	105 (13.6)	77 (11.4)	
	Strongly agree	1266 (87.4)	667 (86.4)	599 (88.6)	
**Reset quit date during quit attempt**					<.001^b^
	No	5032 (81.0)	2757 (84.6)	2275 (77.0)	
	Yes	1183 (19.0)	502 (15.4)	681 (23.0)	
**Days enrolled before starting quit attempt**					<.001^b^
	0	2332 (37.5)	1316 (40.4)	1016 (34.4)	
	1-7	2545 (41.0)	1361 (41.8)	1184 (40.1)	
	8-14	1338 (21.5)	582 (17.9)	756 (25.6)	

^a^*P* value from the Chi-square test.

^b^These values are statistically significant at an alpha level of .05.

^c^Sum does not add to the total due to missing values.

^d^Sum does not add to the total, as users were only given two of eight items at sign up (see Methods section for details).

^e^Time to first cigarette after waking up in the morning.

^f^A little true, a little untrue, or very untrue.

^g^Users were asked about their intention to be smoke free 1 year from signing up.

^h^Agree, disagree, or strongly disagree.

**Figure 1:**
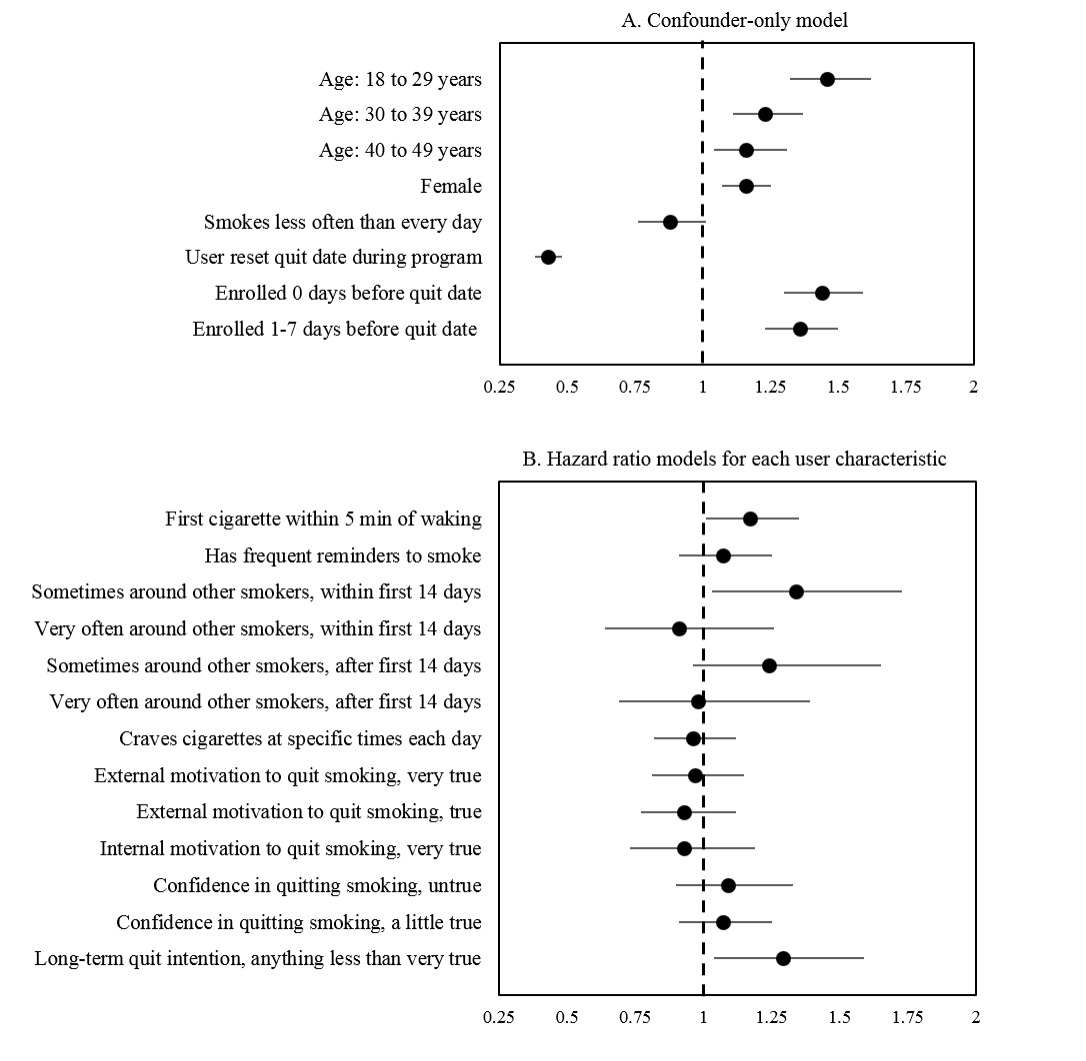
Adjusted survival analysis describing predictors of opting out of SmokefreeTXT, presenting the results of 10 adjusted models. Full model information available is in Multimedia Appendix 2. Panel A presents results from one confounder-only model (age, sex, smoking frequency, reset of quit date by the user, and days enrolled before start of the quit attempt). Panel B presents nine separate survival models with all confounders plus each user characteristic of interest. Violation of the proportional hazards assumption was found for the user characteristic “frequency around other smokers”; models were stratified at 14 days at the point where violation occurred and are presented separately.

Survival analyses revealed that, except for smoking frequency, each variable included in the confounder-only model was associated with opting out (Figure 1, Multimedia Appendix 2). Specifically, women, users younger than 50 years of age, and users who signed up for SmokefreeTXT on their quit date or within 1 week of their quit date were more likely to opt out than others (Figure 1A). Users who reset their quit date after initiating their attempt were less likely to opt out than those who did not reset their quit date. Among the characteristics of interest, people who smoked within 5 minutes of waking up, were sometimes around other smokers, and had lower long-term quit intention were more likely to opt out (Figure 1B). Specifically, users who smoked within 5 minutes of waking were 1.17 times more likely to opt out than those who smoked more than 5 minutes after waking (95% CI 1.01-1.35). Users with less than high long-term intention to be smoke free were 1.29 times more likely to opt out than those with the highest levels of long-term quit intention (95% CI 1.04-1.59). Compared to users who were never or rarely around other smokers, those who were sometimes around other smokers were 1.34 times more likely to opt out during the first 14 days of the program (95% CI 1.03-1.73).

**Figure 2:**
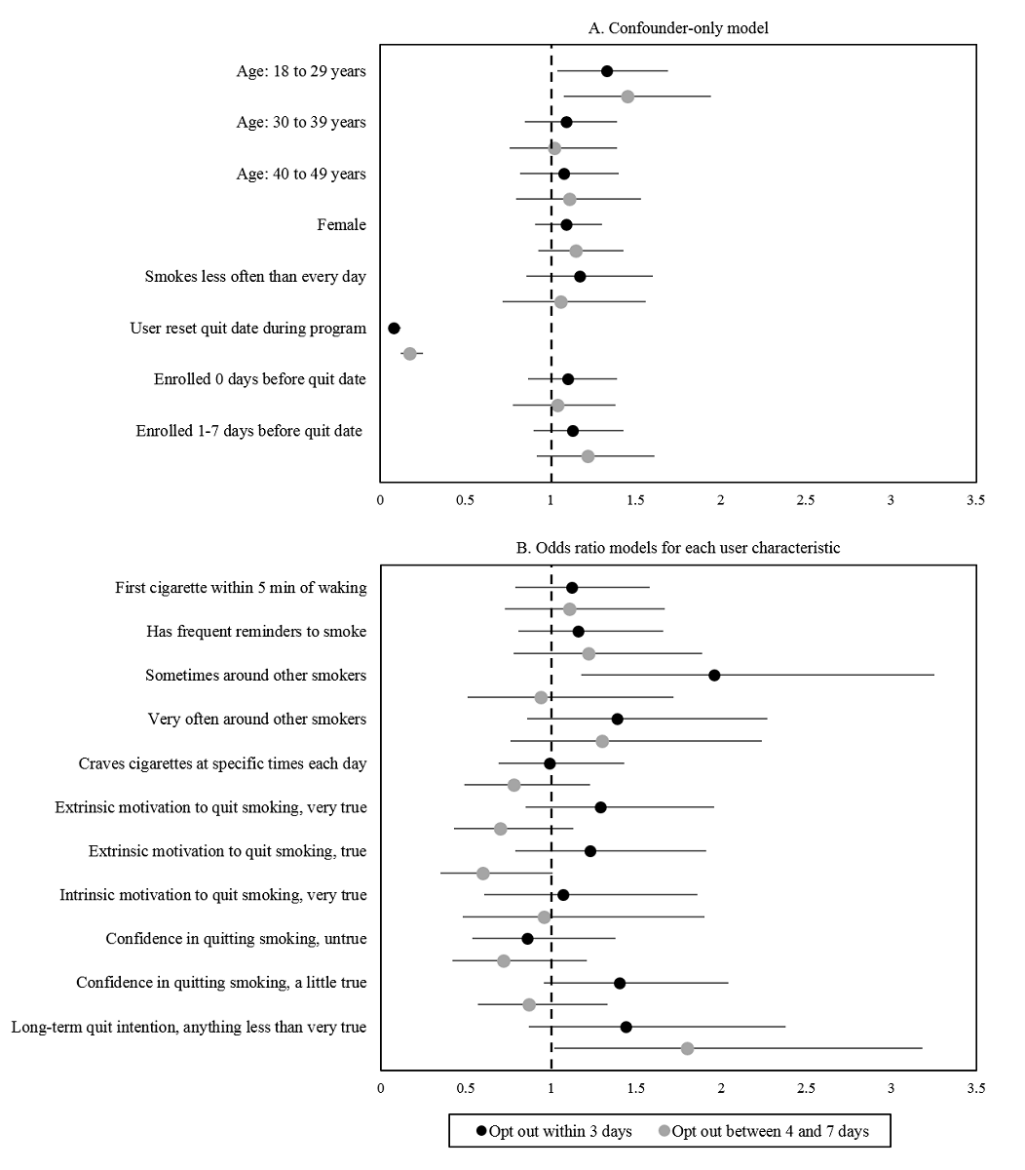
Multivariable multinomial logistic regression model for users who opted out of SmokefreeTXT, comparing users who opted out within 3 days and between 4 and 7 days to those opting out after 7 days. This figure presents results of nine adjusted models. Full model information is available in Multimedia Appendix 3. Panel A presents results from one confounder-only model (age, sex, smoking frequency, reset of quit date by the user, and days enrolled before start of the quit attempt). Panel B presents eight separate logistic regression models with all confounders plus each user characteristic of interest.

Among all users who opted out, several characteristics were associated with opting out early (ie, within the first 3 days of the quit attempt or between 4 and 7 days into the quit attempt vs later; Figure 2, Multimedia Appendix 3). Specifically, compared to users aged ≥50 years, those aged 18-29 years had 1.33 and 1.45 times the odds of opting out within 3 days or between 4 and 7 days into the quit attempt, respectively (95% CI 1.04-1.69 and 95% CI 1.08-1.94, respectively; Figure 2A). Users who reset their quit date after initiating the quit attempt were less likely to opt out of SmokefreeTXT early compared to users who did not reset their quit date (within 3 days: odds ratio 0.08, 95% CI 0.06-0.12; between 4 and 7 days: odds ratio 0.17, 95% CI 0.12-0.25). Compared to users who were never or rarely around other smokers, those who were sometimes around other smokers had 1.96 times the odds of opting out within the first 3 days (95% CI 1.18-3.25; Figure 2B). Lastly, compared to users with the highest levels of long-term quit intention, users with lower levels of long-term quit intention had 1.80 times the odds of opting out between 4 and 7 days into the quit attempt (95% CI 1.02-3.18).

## Discussion

This study assessed smoking context and motivational characteristics associated with opting out of the SmokefreeTXT program in two ways: opting out over the entire cessation intervention and opting out at specific periods early in a cessation attempt. Overall, almost 50% of SmokefreeTXT users were retained in the program over the course of the 6-week intervention. Several user characteristics seemed to influence retention in SmokefreeTXT; these were found to vary in terms of strength of the association and timing relative to the cessation attempt. Specifically, younger age, female sex, and higher levels of nicotine dependence were associated with increased rates of opting out over the entire intervention. Being around other smokers was uniquely associated with opting out early in the quit attempt. Lower long-term intention to stay smoke free was associated with increased rates of opting out over the entire intervention and had a particularly pronounced association with retention in days 4-7 of the quit attempt.

To our knowledge, this is the first study to identify characteristics associated with opting out by using data from a real-world implementation of a text messaging smoking cessation program. Previous analyses of SmokefreeTXT user data found similar rates of opting out among users enrolled between 2012 and 2014 [7]. Therefore, it appears that program changes may be needed to increase retention in this widely used program. The predictors of opting out found in this study are largely consistent with those shown to be predictive of relapse following smoking cessation [17-21], which suggests that users may be leaving the program because they have returned to smoking. This potential pattern of opting out in parallel with relapse is supported by an analysis by Heminger et al [8], who found that participants who opted out of the text messaging smoking cessation program Text2Quit were less likely to be abstinent at 6 months compared with those who did not opt out of the program. It is uncertain how generalizable their results are to the SmokefreeTXT user population, as their analysis occurred in the context of a clinical trial and most participants remained enrolled in the program. The potential differences in how users interact with text-messaging programs within and outside a research setting are potentially large, given that in the study by Heminger et al [8], only 30.2% of participants texted “STOP” [8], yielding a 22% difference between their study and the opt out rates found in the SmokefreeTXT program. To date, there has been no real-world evaluation of the association between program retention and smoking abstinence. The results of this study identified several user characteristics of interest that could be addressed with modifications to the currently available text messaging cessation program.

It is possible that addressing the characteristics associated with opting out might improve retention in the SmokefreeTXT program, which could be done by going beyond the currently available “one-size-fits-all” approach, by potentially including elements of tailoring. Tailoring to specific influential characteristics may increase the relevance of the program to each user and provide more salient tips and resources, thereby increasing retention [22,23]. Previous studies have shown the effectiveness of tailored text message–based cessation interventions [24-26], but to our knowledge, none have been implemented outside a research setting. Further, only one study has compared tailored to nontailored text messaging cessation programs [27]. Thus, the full potential impact of tailoring within text messaging cessation programs and the features requiring tailoring the most are unknown. The results of the survival analysis provide information about the characteristics of users who had significantly higher rates of opting out over the entire 42-day SmokefreeTXT program, highlighting several characteristics that could be the focus of tailoring throughout text messaging–based cessation programs.

Users younger than 50 years of age were more likely to opt out of SmokefreeTXT. Further, those aged 18-29 years had the highest risk of opting out over the entire intervention and early in the quit attempt. Previous research among young adult smokers have identified several unique features that may make quitting more difficult in this group. For example, there is evidence that young adults’ identity as smokers continues to evolve [28] and young adults who smoke frequently might still not identify as smokers [29-33]. This could be a result of younger smokers having lower rates of daily smoking compared to older adult smokers [1]. It is therefore possible that even while making a quit attempt, traditional cessation programs might not resonate with young adult smokers. Previous evaluations of motivations to quit smoking among young adult smokers have identified physical fitness and worry about long-term health effects of smoking as motivators to quit [34,35]. Thus, incorporating additional intervention content in these areas may assist in keep young adult smokers engaged in a cessation program. In addition, it is possible that identifying ways to help reinforce a young adult’s identity as a smoker could maintain motivation to quit in order to avoid the long-term health harms already associated with smoking [36]. Lastly, young adults have the highest rates of technology adoption [37]. Although a text messaging program might align with how young adults are using devices, it may be harder for an intervention to resonate with high-technology adopters if it does not include the most up-to-date functionality. Continued exploration of the barriers to quitting and remaining engaged in a cessation attempt among this group is imperative to provide up-to-date, relevant content within cessation programs.

Logistic regression analyses identified two characteristics found to be particularly important early in the quit attempt: being around other smokers sometimes and having low long-term quit intention. However, the timing of these associations differed, even within the first week of the quit attempt. Including messages to address these characteristics within the first days of a quit attempt may increase retention. Frequency of being around other smokers can be an indicator of how frequently a smoker initiating a cessation attempt might experience cues and urges to smoke, which can derail a quit attempt [38-40]. Being around other smokers may also present a perceived smoking opportunity to smokers undertaking a quit attempt, which can also increase cravings to smoke [41]. In this study, compared to users who were never around other smokers, only those who were sometimes around other smokers had higher odds of opting out. There was no increased risk of opting out for users who were frequently around other smokers, compared to users who were never around other smokers. This finding could indicate that people who are only sometimes around other smokers (almost one-third of SmokefreeTXT users) face unexpected cravings that they are not prepared for and indicates the potential need for additional skill building early in the quit attempt to help plan for and resist unexpected cravings. One way to do this could be to provide additional reminders about the on-demand keyword functionality embedded within text message programs to people who are only intermittently around other smokers in order to assist with cravings and increase the use of real-time support during their quit attempt.

People with lower levels of long-term quit intention had an increased risk of opting out over the entire intervention and had higher odds of opting out between days 4 and 7 of the quit attempt. This finding is consistent with the Theory of Planned Behavior (TPB), which suggests that without intention, subsequent behavioral changes will not occur [15]. A previous meta-analysis determined that the TPB applies to smoking behavior, specifically that smoking intentions predict smoking behavior and that the key constructs of the TPB (attitudes, subjective norms, and perceived behavioral control) were all associated with smoking intentions [42]. Health behavior theories like the TPB are promoted for use in interventions to change addictive behaviors [43-45]. Using the TPB by incorporating additional strategies to change social norms, attitudes, and perceived behavioral control may help maintain engagement of smokers undergoing a quit attempt.

Users who smoked within 5 minutes of waking were more likely to opt out prior to program completion compared to those who reported a longer interval to their first cigarette of the day. Time to first cigarette is an indicator of the level of nicotine dependence [12], and smokers with higher levels of nicotine dependence are more likely to relapse [46,47]. The level of nicotine dependence represents a potentially important dimension for treatment tailoring; smokers with higher versus lower levels of nicotine dependence might benefit from different forms, doses, or schedules of pharmaceutical support to achieve long-term smoking cessation. For example, evaluations of pharmacotherapy for cessation show that smokers with higher levels of nicotine dependence benefit more from combination pharmacotherapy (ie, combinations of smoking cessation medications) than smokers with lower levels of nicotine dependence. Currently, nicotine replacement therapy (NRT) or other pharmaceutical cessation aids are not provided to SmokefreeTXT users. Nonetheless, there is potential opportunity to increase the use of NRT, particularly among SmokefreeTXT users with the highest levels of nicotine dependence, by tailoring the intervention to provide additional suggestions and information on the use of NRT in this group. Additionally, the use of pharmaceutical therapies for cessation is higher among former smokers who visited a physician in the year before they quit smoking [48]; thus, identifying ways to integrate clinical care, where access to pharmaceuticals is increased, and digital cessation care might also lead to more successful quitting attempts and motivation to stay engaged with a text message–based cessation program. One example of integration of clinical and digital cessation is the use of the electronic health record to identify patients who currently smoke, which then prompts providers to offer tobacco dependence counseling and medication and include a referral to the local quit line [49,50]. Within this framework, it could also be feasible for clinical practices to include referral to other digital tools like text messaging, depending on patient preference.

People who enrolled within 1 week of starting their quit attempt were more likely to opt out than those who enrolled between 1 and 2 weeks before initiating their quit attempt. One interpretation of this result is that greater exposure to preparation messages increased the odds of successfully quitting smoking. This finding is in line with clinical practice guidelines, which recommend that smokers create a quit plan and take time to prepare for their quit attempt [51]. Previous text messaging–based cessation interventions have also included time for preparation by requiring participants to set quit dates in the future [26,52,53]. However, when implemented in a real-world setting, there is no oversight about how much preparation a user receives and in our study population, almost 38% of users opted to initiate their quit attempt immediately and thus received no preparation messages. Further, requiring a specific amount of preparation may create a barrier to accessing support for a smoker who is ready to quit immediately. Since SmokefreeTXT users are allowed to set their own quit date within a 2-week period, it is unclear what the impact would be if all users received the same amount of preparation messages. Further, it is unknown how users are selecting their initial quit date in this setting. Future research should examine the motivations and preferences of users setting their own quit dates and continue to explore this critical period of the cessation attempt.

People who used the feature of resetting their quit date were less likely to opt out of SmokefreeTXT. Use of this feature was the only independent variable to show a protective effect with opting out and was associated with early and overall program retention. It is possible that use of this functionality represents smokers remaining committed to their cessation goals, especially as use of this feature is optional. Staying committed to a quit attempt is extremely important early on when the risk of smoking relapse is the highest; therefore, utilization of this feature could represent people who might have left after failing to initiate their quit attempt (ie, smoked on quit day), but instead of giving up on quitting smoking altogether and opting out, they immediately recommitted to quitting by using the reset feature. Thus, there is potential value in reframing a smoking lapse not as a failure but as part of the process of cessation that can still help smokers get to their end goal of being smoke free. Further investigation is needed to better understand this phenomenon and the best way to promote re-engagement in a cessation attempt after a slip or another setback.

This study has several limitations that need to be considered. First, given the real-world implementation of this study, participant burden needed to be minimized; therefore, only two additional baseline items were added per user to the SmokefreeTXT sign up webpage. As a result, complete information for all the independent variables of interest were not available for each user. However, as these data are all missing completely at random, future research could consider employing imputation techniques to allow for simultaneous examination of all independent variables of interest. Second, to reduce respondent burden, only single-item measures were used for data collection. However, validated items and scales were used when available. Additionally, this study focused on smoking context and motivational characteristics associated with retention and therefore did not measure all potential user characteristics of interest, such as measures of depression or stress, or other substance use behaviors like alcohol use. Fortunately, it appears that SmokefreeTXT users tolerated the additional items, as sign-up rates to the program were not reduced during the study. Therefore, a future implementation could consider adding more than two new items to the sign up webpage to expand data collection. Lastly, this study was unable to account for the potential influence of technology-related characteristics; for example, previous use of text-messaging programs and cell phone availability during working hours or assessment of users’ input on why they decided to opt out. Future qualitative studies might be particularly well suited to explore the larger contextual factors that impact a smokers’ use and disuse of technology-based interventions.

This study identified several important smoking context and motivational characteristics associated with opting out of a smoking cessation text-messaging program. Although some characteristics were associated with opting out over the entire 42-day cessation program, others were only influential very early into the quit attempt. The user characteristics and programmatic features found to be associated with opting out could be addressed through program changes, such as tailoring of new messages or integration of mHealth tools with clinical care. For example, program changes to incorporate tailoring for characteristics like age, frequency of being around other smokers, and motivations to quit smoking might increase the salience of the program to users. Integration with clinical care could help support smokers with higher levels of nicotine dependence by facilitating use of NRTs and other pharmaceutical cessation aids. These modifications, in combination with promoting the use of preparation messages and helping users remain committed to quitting smoking, could increase retention and engagement, which could ultimately increase success in quitting smoking.

## Appendix

Multimedia Appendix 1 User characteristics by the timing of the opt-out variable.

Multimedia Appendix 2 Adjusted survival analysis describing predictors of opting out of SmokefreeTXT.

Multimedia Appendix 3 Characteristics associated with opting out early.

## Abbreviations

mHealth: mobile health
NRT: nicotine replacement therapy
TPB: Theory of Planned Behavior
