# Supplementary material for: Predictors of Retention in an Adult Text Messaging Smoking Cessation Intervention Program: Cohort Study
Source: JMIR Mhealth Uhealth. 2019 Aug 1;7(8):e13712. doi: 10.2196/13712 (PMC6694733; doi:10.2196/13712)
Supplement: Multimedia Appendix 1 [file mhealth_v7i8e13712_app1.pdf]

Multimedia Appendix 1. Descriptive frequencies by timing of opt out among those who opted out (N=3259).

Variable		Opt out timing			p-value[a]
		Within 3 days n (%)	Between 4 and 7 days n (%)	After 7 days n (%)	
Age					
	18 to 29 years	356 (34.7)	205 (36.6)	491 (29.4)	0.02[b]
	30 to 39 years	298 (29.0)	150 (26.8)	509 (30.4)	
	40 to 49 years	202 (19.7)	113 (20.2)	344 (20.6)	
	50 + years	171 (16.6)	92 (16.4)	328 (19.6)	
Sex					
	Male	304 (29.6)	158 (28.2)	489 (29.3)	0.84
	Female	723 (70.4)	402 (71.8)	1183 (70.7)	
Smoking frequency[c]					
	< Every day	80 (7.8)	39 (7.0)	112 (6.8)	0.59
	Every day	940 (92.2)	518 (93.0)	1534 (93.2)	
Time to first cigarette[d,e]					
	> 5 minutes	145 (58.7)	83 (58.9)	283 (62.7)	0.50
	≤ 5 minutes	102 (41.3)	58 (41.1)	168 (37.3)	
Frequent reminders to smoke[d]					
	Not true[f]	77 (31.1)	39 (29.1)	136 (32.5)	0.76
	Very true	171 (68.9)	95 (70.9)	283 (67.5)	
Frequency around other smokers[d]					
	Never or rarely	35 (14.4)	26 (17.4)	84 (20.4)	0.01[b]
	Sometimes	94 (38.7)	36 (24.2)	117 (28.5)	
	Very often	114 (46.9)	87 (58.4)	210 (51.1)	
Craves cigarettes at a specific time of day[d]					
	No	182 (68.2)	95 (72.0)	259 (66.4)	0.49
	Yes	85 (31.8)	37 (28.0)	131 (33.6)	
Extrinsic motivation to quit[d]					
	Very true	112 (44.5)	51 (38.6)	184 (42.5)	0.07
	A little true	84 (33.3)	34 (25.8)	141 (32.6)	
	A little or very untrue	56 (22.2)	47 (35.6)	108 (24.9)	
Intrinsic motivation to quit[d]					
	Not true[f]	28 (9.6)	13 (10.0)	38 (9.6)	0.99
	Very true	265 (90.4)	117 (90.0)	357 (90.4)	
Confidence in quitting smoking[d]					
	A little or very untrue	43 (17.4)	29 (18.1)	91 (21.4)	0.23
	A little true	118 (47.8)	62 (38.8)	174 (40.8)	
	Very true	86 (34.8)	69 (43.1)	161 (37.8)	
Long-term quit intention[d,g]					
	Other responses[h]	35 (14.8)	24 (17.9)	46 (11.5)	0.14
	Strongly agree	202 (85.2)	110 (82.1)	355 (88.5)	
Reset quit date during quit attempt					
	No	997 (97.1)	527 (94.1)	1233 (73.7)	<.001[b]
	Yes	30 (2.9)	33 (5.9)	439 (26.3)	
Days enrolled before starting quit attempt					

	0 days	430 (41.9)	219 (39.1)	667 (39.9)	0.54
	1-7 days	423 (41.2)	246 (43.9)	692 (41.4)	
	8-14 days	174 (16.9)	95 (17.0)	313 (18.7)	

[a]P-value from Chi-square test

[b]These values are statistically significant at an alpha level of 0.05.

[c]Sum does not add to total due to missing.

[d]Sum does not add to total as users were only given two of eight items at sign-up. See Methods section for details.

[e]Time to first cigarette after waking up in the morning

[f]A little true, a little untrue, or very untrue

[g]Users asked intention to be smoke free one year from signing up

[h]Agree, disagree or strongly disagree
