# Supplementary material for: Predictors of Retention in an Adult Text Messaging Smoking Cessation Intervention Program: Cohort Study
Source: JMIR Mhealth Uhealth. 2019 Aug 1;7(8):e13712. doi: 10.2196/13712 (PMC6694733; doi:10.2196/13712)
Supplement: Multimedia Appendix 2 [file mhealth_v7i8e13712_app2.pdf]

Multimedia Appendix 2. Adjusted survival analysis describing predictors of opting out of SmokefreeTXT

Variable			HR (95% CI)
**Confounder-only model (N=6140)**			
	Age		
		18 to 29 years	1.46 (1.32-1.62)[a]
		30 to 39 years	1.23 (1.11-1.37)[a]
		40 to 49 years	1.16 (1.04-1.31)[a]
		50 + years	Referent
	Sex		
		Male	Referent
		Female	1.16 (1.07-1.25)[a]
	Smoking frequency		
		< Every day	0.88 (0.76-1.01)
		Every day	Referent
	User reset quit date during program		
		No	Referent
		Yes	0.43 (0.38-0.48)[a]
	Days enrolled before starting quit attempt		
		0 days	1.44 (1.30-1.59)[a]
		1-7 days	1.36 (1.23-1.50)[a]
		8-14 days	Referent
**Hazard ratio models for each user characteristic**			
	Time to first cigarette (n=1613)[b,c]		
		> 5 minutes	Referent
		≤ 5 minutes	1.17 (1.01-1.35)[a]
	Frequent reminders to smoke (n=1490)[b]		
		Not true[d]	Referent
		Very true	1.07 (0.91-1.25)
	Frequency around other smokers within 14 days (n=518)[b,e]		
		Never or rarely	Referent
		Sometimes	1.34 (1.03-1.73)[a]
		Very often	1.24 (0.97-1.59)
	Frequency around other smokers after 14 days (n=1037)[b,e]		
		Never or rarely	Referent
		Sometimes	0.91 (0.63-1.32)
		Very often	0.98 (0.69-1.39)
	Craves cigarettes at a specific time of day (n=1507)[b]		
		No	Referent
		Yes	0.96 (0.82-1.12)
	Extrinsic motivation to quit (n=1572)[b]		
		Very true	0.97 (0.81-1.15)
		A little true	0.93 (0.77-1.12)
		A little or very untrue	Referent
	Intrinsic motivation to quit (n=1508)[b]		
		Not true[d]	Referent
		Very true	0.93 (0.73-1.19)
	Confidence in quitting smoking (n=1533)[b]		

		A little or very untrue	1.09 (0.90-1.33)
		A little true	1.07 (0.91-1.25)
		Very true	Referent
	Long-term quit intention (n=1428)[b,f]		
		Other responses[g]	1.29 (1.04-1.59)[a]
		Strongly agree	Referent

Note: HR = Hazard ratio, CI= Confidence interval. Table presents results of ten adjusted models. One for the confounder only model (age, sex, smoking frequency, if user reset quit date, and days enrolled before start of the quit attempt) and nine separate survival models with all confounders plus each user characteristic of interest.

[a]These values are statistically significant at an alpha level of 0.05.

[b]Model adjusted for age, sex, smoking frequency, if user reset quit date, and days enrolled before start of quit attempt.

[c]Time to first cigarette after waking up in the morning

[d]A little true, a little untrue, or very untrue

[e]Violation of proportional hazards assumption found for the user characteristic "frequency around other smokers". Models stratified at 14 days at the point where violation occurred.

[f]Users asked intention to be smoke free one year from signing up

[g]Agree, disagree or strongly disagree
