# Supplementary material for: Predictors of Retention in an Adult Text Messaging Smoking Cessation Intervention Program: Cohort Study
Source: JMIR Mhealth Uhealth. 2019 Aug 1;7(8):e13712. doi: 10.2196/13712 (PMC6694733; doi:10.2196/13712)
Supplement: Multimedia Appendix 3 [file mhealth_v7i8e13712_app3.pdf]

Multimedia Appendix 3. Multivariable multinomial logistic regression model among users who opted out of SmokefreeTXT comparing users who opted out within 3 days, and between 4 and 7 days, to those opting out after 7 days.

			Opt out timing	
**Variable**			**Within 3 days**	**Between 4-7 days**
**Confounder-only model (N=3223)**			**OR (95% CI)**	**OR (95% CI)**
	Age			
		18 to 29 years	1.33 (1.04-1.69)[a]	1.45 (1.08-1.94)[a]
		30 to 39 years	1.09 (0.85-1.39)	1.02 (0.76-1.39)
		40 to 49 years	1.08 (0.82-1.40)	1.11 (0.80-1.53)
		50 + years	Referent	Referent
	Sex			
		Male	Referent	Referent
		Female	1.09 (0.91-1.30)	1.15 (0.93-1.43)
	Smoking frequency			
		< Every day	1.17 (0.86-1.60)	1.06 (0.72-1.56)
		Every day	Referent	Referent
	User reset quit date during program			
		No	Referent	Referent
		Yes	0.08 (0.06-0.12)[a]	0.17 (0.12-0.25)[a]
	Days enrolled before starting quit attempt			
		0 days	1.10 (0.87-1.39)	1.04 (0.78-1.38)
		1-7 days	1.13 (0.90-1.43)	1.22 (0.92-1.61)
		8-14 days	Referent	Referent
**Logistic regression models for each user characteristic**				
	Time to first cigarette (n=825)[b,c]			
		> 5 minutes	Referent	Referent
		≤ 5 minutes	1.12 (0.79-1.58)	1.11 (0.73-1.67)
	Frequent reminders to smoke (n=789)[b]			
		Not true[d]	Referent	Referent
		Very true	1.16 (0.81-1.66)	1.22 (0.78-1.89)
	Frequency around other smokers (n=798)[b]			
		Never or rarely	Referent	Referent
		Sometimes	1.96 (1.18-3.25)[a]	0.94 (0.51-1.72)
		Very often	1.39 (0.86-2.27)	1.30 (0.76-2.24)
	Craves cigarettes at a specific time of day (n=779)[b]			
		No	Referent	Referent
		Yes	0.99 (0.69-1.43)	0.78 (0.49-1.23)
	Extrinsic motivation to quit (n=810)[b]			
		Very true	1.29 (0.85-1.96)	0.70 (0.43-1.13)
		A little true	1.23 (0.79-1.91)	0.60 (0.35-1.01)
		A little or very untrue	Referent	Referent
	Intrinsic motivation to quit (n=811)[b]			
		Not true[d]	Referent	Referent
		Very true	1.07 (0.61-1.86)	0.96 (0.48-1.90)
	Confidence in quitting smoking (n=825)[b]			
		A little or very untrue	0.86 (0.54-1.38)	0.72 (0.42-1.21)
		A little true	1.40 (0.96-2.04)	0.87 (0.57-1.33)

		Very true	Referent	Referent
	Long-term quit intention (n=763)[b,e]			
		Other responses[f]	1.44 (0.87-2.38)	1.80 (1.02-3.18)[a]
		Strongly agree	Referent	Referent

Note: OR = Odds ratio, CI = Confidence interval. Outcome referent = opted out after 7 days into the quit attempt. Table presents results of nine adjusted models. One for the confounder only model (age, sex, smoking frequency, if user reset quit date, and days enrolled before start of quit attempt) and eight separate logistic regression models with all confounders plus each user characteristic of interest.
[a]These values are statistically significant at an alpha level of 0.05.

[b]Model adjusted for age, sex, smoking frequency, if user reset quit date, and days enrolled before start of quit attempt.

[c]Time to first cigarette after waking up in the morning

[d]A little true, a little untrue, or very untrue

[e]Users asked intention to be smoke free one year from signing up

[f]Agree, disagree or strongly disagree
